# Bidirectional Relationship Between Family Accommodation and Youth Anxiety During Cognitive-Behavioral Treatment

**DOI:** 10.1007/s10578-021-01304-5

**Published:** 2022-01-08

**Authors:** Thomas B. Bertelsen, Joeseph A. Himle, Åshild Tellefsen Håland

**Affiliations:** 1 Department of Child and Adolescence Mental Health, Sørlandet Sykehus, Kristiansand, Norway; 2 Department of Clinical Child and Adolescent Psychology, Faculty of Psychology, University of Bergen, Bergen, Norway; 3 School of Social Work and Department of Psychiatry, University of Michigan, Ann Arbor, MI USA

**Keywords:** Family accommodation, Anxiety, Youth, CBT, Mechanisms

## Abstract

Family accommodation is associated with an increase in anxiety and has recently received attention as a target for intervention for youth anxiety. Existing theories posit that the increase in family accommodation increases youth anxiety and can attenuate the effect of psychotherapy. However, the directionality between family accommodation and youth anxiety has not been investigated. A cross-lagged cross-panel design was used to assess accommodation and anxiety for 10 sessions for 73 youths with an anxiety disorder, who were receiving cognitive-behavioral therapy. The analysis revealed a bidirectional relationship, such that to some extent previous session family accommodation increased youth anxiety symptoms (*β* = 0.11, 95% CI [0.06, 0.17]), but to an even greater extent previous session youth-rated anxiety symptoms increased family accommodation (*β* = 0.23, 95% CI [0.08, 0.38]). Family accommodation is an important target for reducing youth anxiety but should be addressed simultaneously as interventions directly targeting youth anxiety.

## Introduction

Anxiety disorders are among the most common mental health issues of children and adolescents [1]. Without treatment, many youth with anxiety disorders will continue to experience these and other psychiatric conditions into adulthood [2]. These disorders impair cognitive development, school performance, and social functioning of afflicted youth [3–5], and present with high rates of co-occurring psychiatric conditions, such as depression, addiction and heightened risk of suicidal behavior [6, 7]. These disorders are also associated with school absenteeism, parental productivity loss, and increased healthcare service usage, and thus present a substantial societal burden [8].

Recently, the term “family accommodation” has been identified as a potentially important target for interventions aimed at reducing youth anxiety [9, 10]. Family accommodation is defined as any change that parents make to their behavior with the intent of lessening or protecting their child from anxiety or fear in the short-term [11, 12]. Current theories posit that family accommodation increases anxiety over time through negative reinforcement of avoidance behaviors that interfere with the natural extinction of conditioned fear [9]. Family accommodation may be seen as a part of a broader set over overprotective parenting behaviors, which include intrusive parental involvement and reduced autonomy for the child [11]. Such behaviors are believed to maintain youth anxiety due to reduced self-efficacy, which causes the youth to avoid novel situations that could offer opportunities for mastery of their anxiety [13, 14]. Based on this theoretical model, treatments that aim to reduce family accommodation have been developed and have shown effectiveness in reducing anxiety [12, 15]. Although such treatments are promising, the theorized directional relationship between family accommodation and anxiety has not been sufficiently investigated.

Previous research on youth anxiety and family accommodation has examined the association between the two and found that family accommodation plays a mediating role in child functional impairment due to anxiety [16, 17]. Further research has established that family accommodation and youth anxiety are positively correlated, and that a reduction in family accommodation is associated with a reduction in anxiety symptoms [18–20]. A limitation of previous research is that it has not sufficiently determined to what extent reduced anxiety symptoms precede reduced family accommodation, which is also a plausible explanation for the relationship between these two factors [19, 21]. To deliver effective interventions to youth with anxiety disorders, it is important to understand the directional relationship between family accommodation and anxiety symptoms. If change in anxiety symptoms precedes change in family accommodation, resources should be mostly allocated to target anxiety and less toward family accommodation (see [22]). Conversely, if change in family accommodation precedes change in anxiety symptoms, emphasis should be placed on resources aimed at reducing family accommodation (see [23]).

The current study assessed the directional relationship between family accommodation and youth anxiety in youth undergoing treatment by using a multilevel bivariate autoregressive cross-lagged model [24]. In addition to analyzing directional relationships between variables, this model accounts for individual differences, which increases the generalizability of findings [24, 25]. To understand the directional relationship between family accommodation and youth anxiety we investigated the directional relationship from the previous session to the current session. We hypothesized that increased accommodation would precede increased anxiety and that increased anxiety precedes in increased family accommodation. We also hypothesized that the effect of accommodation on anxiety would be stronger than the effect of anxiety on accommodation.

## Methods

### Participants

The participants were part of a study assessing the effectiveness of group-based CBT with family and school involvement in a community clinic. More detailed information on the study will be available in [26], and thus the present article will only give a brief overview of study methods. Participants were recruited from two community clinics for child and adolescent mental health between 2017 and 2019. These clinics serve a population of 76,000 children and youth under 18 years, in both rural and urban areas of southern Norway. The inclusion criteria for participation were meeting DSM-IV [27] for a primary anxiety diagnosis (i.e., separation anxiety disorder, social anxiety disorder, specific phobia, panic disorder with or without agoraphobia or generalized anxiety disorder) as assessed by the Anxiety Diagnostic Interview Schedule child and parent version (ADIS-C/P; [28]). Potential participants were excluded if they met criteria for a developmental or psychotic disorder, if they had ongoing self-harm behavior or suicidal ideation. Further exclusion criteria were as follows: concurrent participation in psychological treatment, a psychopharmacological treatment that had not been stable for 6 months before study enrolment and receiving cognitive-behavioral therapy within past 12 months. Additionally, participants were required to be attending school more than 50% of the time over the previous month. The final requirement was due to practical concerns regarding the involvement of school personnel in treatment. Further details on exclusion and inclusion criteria are to be found in [26]. In the recruited sample, 17 youths with a diagnosis of OCD also participated. These were excluded from the current study to focus specifically on anxiety disorders. The Regional Ethics Committee for research with human subjects approved the study (reg. nr. 2017/1367) and written informed consent was obtained from the entire sample before inclusion.

Participants were 73 youths, aged 12–18 (*M* = 15.4, *SD* = 1.4, 75% female) and at least one accompanying parent (43.8% had two accompanying parents at first session). The primary diagnoses for the included youth were the following: social anxiety disorder (69.9%), agoraphobia or panic disorder or both (10.9%), generalized anxiety disorder (10.9%), separation anxiety disorder (5.5%), and specific phobia (2.7%). Comorbid conditions included the following: generalized anxiety disorder (45.2%), specific phobia (21.9%), social anxiety disorder (9.5%), agoraphobia or panic disorder or both (9.5%), major depressive disorder (9.5%), separation anxiety disorder (6.8%), Tourette’s syndrome (2.7%) and attention deficit hyperactivity disorder (1.3%). All participating youths were ethnic Norwegian and attending school.

### Treatment

The treatment comprised 12 sessions conducted over 10 weeks, with the first and the last session only including parents and school personnel. Four clinicians conducted each session with 5–8 youths and their accompanying parents participating. Data were gathered on the 10 sessions where youth were attending and not the two sessions where only parents and school personnel participated. The 10 sessions where youths attended lasted between 2.5 [6 sessions] and 5 h (4 sessions), with a total of 35 h of treatment. In all sessions, parents and youth spent some time together in a large group and also some time separated into a youth-group and a parent-group. In the shorter sessions, the focus in the youth-only portions of the sessions, focus was on collaboratively planning and conducting exposure practice. In the parent-only portions of the sessions, emphasis included parents collaboratively reflecting on parent accommodation and/or punishing behavior and making plans on how to change these unproductive behaviors. The longer sessions were dedicated to maximizing time spent performing exposure practice and included at least 3 h of therapist/family/peer-facilitated exposure practice. Exposure practice consisted of gradual exposure to the feared situation or stimuli and was adapted to the individual youth. Thus, the treatment included a high degree of parental involvement with a focus on reducing family accommodation. Additionally, the treatment included involvement of school-personnel, but the amount and type of involvement varied based on the needs of individual youth.

### Therapist and Therapist Training

During the study, twenty clinicians participated with a mean of 11.8 years of experience in child and adolescent mental health care (*SD* = 7.9, range = 2–30). Clinicians were employed at the participating clinics and included six clinical psychologists, six social workers, four nurses specialized in psychiatry, two child psychiatrists, one pediatrician, and one schoolteacher with training in mental health.

### Diagnostic Interview

DSM-IV anxiety disorder diagnoses were established using the *Anxiety Disorders Interview Schedule–Children and Parent versions* (ADIS-C/P; [29]), administered separately to parents and their youth by clinicians trained in its use. ADIS-C/P is a semi-structured interview with excellent reliability for diagnoses (Cronbach’s α = 0.80–0.92) and strong correspondence with anxiety questionnaires [29]. The ADIS-C/P was administered by clinicians participating in the intervention who were trained in its use by a licensed ADIS-C/P rater. From the recruited sample, which included participants with OCD, a subset of 20% of the interviews was independently rated. The inter-rater reliability between clinicians and independent raters was high (Cronbach’s α = 0.86) for primary diagnosis.

### Measures

For the outcome measures, we assessed parent reported family accommodation and youth reported anxiety symptoms. The use of both parents and youth as informants was done to avoid *confirmation bias* [30], which may have occurred if only a single informant was used. An example of this bias would be if parents decreased accommodation and expected this decrease to lower their child's anxiety symptoms. If the parents then reported lower anxiety symptoms in their child, it would be uncertain whether the parents were simply confirming what they believed ought to occur or whether a real change had occurred. In previous literature on family factors and child anxiety, such biases have been noted [21]. To avoid this, we used multiple informants with ratings on accommodation reported by parents and ratings of youth anxiety symptoms reported by youths.

#### Family Accommodation Scale—Anxiety

The Family Accommodation Scale—Anxiety (FASA; [31]) is the most widely used instrument for assessing family accommodation of child anxiety symptoms. FASA comprises 13 parent-rated items rated on a 5-point Likert-type scale from 0 (“no accommodation or never”) to 4 (“daily”). FASA has strong psychometric properties, including convergent and divergent validity, and internal consistency with Cronbach’s α of 0.90 [31], and test–retest reliability [32]. The FASA total accommodation score was used and internal consistency in the current study was high (Cronbach’s α = 0.92). When both parents had completed the FASA in a given session (36.1%) the mean of the two ratings were used. Otherwise, mother-rated FASA (46.7%) and father-rated FASA (9.5%) were used. The inter-rater agreement between parents was high (Cronbach’s α = 0.82), which indicated that combining mother- and father-ratings was reasonable.

#### Spence Anxiety Scale for Children—Short Form

The Spence Anxiety scale for children—short form [33] (SCAS-C-S) was used to assess youth anxiety severity. The SCAS-C-S is a psychometrically sound short form of the well-established SCAS-C. The short form comprises 8 items that assess the occurrence of anxiety symptoms in the last week rated on a four-point scale (0 = never; 1 = Sometimes; 2 = Often; 3 = Always). The SCAS-C-S has good reliability and internal consistency [33]. We modified the short version of the SCAS adding a single question assessing how much children have avoided feared situations or objects during the previous week, ranging from (0 = Not at all, 1 = Sometimes, 2 = Often, 3 = Very much). In this study, the SCAS-C-S showed good internal consistency (Cronbach’s α = 0.78).

### Procedure

At the end of each session, participants filled out the process measures FASA and SCAS. These process measures assess family accommodation and anxiety symptoms over the previous week. Since the treatment included sessions conducted in the same week (sessions 4 and 5, and 8 and 9), participants were instructed to base their answers on experiences since the previous session. The decision to fill out process measures at the end of the session was done primarily for pragmatic reasons, since clinicians and participants showed a preference for this. However, it is important to note that this may have caused respondents to base answers on experiences from the current session.

### Data Analytical Strategy

Descriptive analyses, bivariate correlations, and linear regressions were performed using the R software [34]. The directional relationship between family accommodations was investigated by inspecting the cross-lagged effects in the multilevel bivariate autoregressive cross-lagged model [24]. A cross-lagged effect refers to the relationship between two variables measured once and then again later. By comparing the strength of the relationship between each variable at the first point in time with the other variable at the second point in time, one can conclude directional relationship between the two variables [24]. When such analysis indicates that one variable precedes the other it can be said to be *Granger-causal* [35]. Granger-causality does not imply causality in the philosophical sense, but in practical terms it may still be highly useful to understand whether change in one variable precedes change in another. Cross-lagged effects were investigated using a Bayesian multivariate cross-lagged model implemented in the R package brms [36], with non-informed priors. A Bayesian approach was preferred since it allowed a flexible model specification and accounted for the uncertainty of all modelled parameters [24]. Covariates that did not vary across time were not included to avoid unnecessary model complexity. All variables were person-mean centered to allow interpretation at the individual level.

The sample size was adequate based on studies suggestions by [37]. Based on simulations, we expected a lower limit of 80% power to detect effects, given the following assumptions: Intraclass correlation coefficient was less than 0.2, a magnitude of cross-lagged associations greater than 0.07, and magnitude in the difference of cross-lagged associations greater than 0.05.

The model was specified using non-informed priors and estimated using Hamiltonian no U-turn estimation with 10.000 iterations across 3 chains in the brms package [36]. The model converged based on Gelman-Rubin statistics, $\widehat{R,}$ and the effective sample size. The fit of the model was evaluated using the Bayes *R*^*2*^ [38] and assessed with posterior predictive checks. The interpretation of the model was based on 95% credibility intervals from the posterior sample. Where the 95% credible interval of an estimate spanned zero, the effect was considered non-substantial. Comparison of cross-lagged associations was done by investigating the posterior probability that one effect was larger than the other based on the Savage-Dickey ratio [39].

Overall, there was 22.6% missing data in the two variables studied, corresponding to sessions where participants were not present. Individuals who had disengaged from treatment after 1–2 sessions accounted for 19.7% of the missing data. Based on visual inspection and analysis of the data, we assumed the data was missing at random. Following existing literature [40], we assumed that multiple imputation using 10 imputed samples for 10,000 iterations would lead to unbiased estimates, given that the model for missing variables was highly efficient (*R*^2^ = 0.50). As a control, analyses were also performed without imputation. Although the magnitude of directional relationships decreased with the unimputed data the conclusions reached were identical to those reached when analyzing using imputed data. Thus, we only present here the analysis based on imputed data.

## Results

The distribution and bivariate correlations across time between parent-rated family accommodation and youth-rated anxiety symptoms are summarized in Table 1. As expected, there was autocorrelation from session to session for FASA (ρ = 0.24) and SCAS (ρ = 0.17). Somewhat surprisingly, there was no significant correlation (*p* > 0.05) between SCAS and FASA within the same session. That is, within the same session parent-rated family accommodation and youth-rated anxiety are not related.

**Table 1:** Correlations between youth anxiety and family accommodation across treatment

Session no	FASA		SCAS		SCAS × FASA
	*M*	*SD*	*M*	*SD*	*r*
Session 1	13.15	8.54	14.00	4.39	− 0.06
Session 2	11.47	7.57	12.53	4.33	− 0.12
Session 3	11.08	8.03	11.21	3.83	0.01
Session 4	8.69	7.00	10.40	3.86	− 0.08
Session 5	8.03	6.79	9.80	3.98	− 0.01
Session 6	9.55	8.19	9.52	3.98	− 0.09
Session 7	8.53	7.07	9.97	4.09	0.16
Session 8	7.24	5.98	9.08	4.01	0.19
Session 9	6.94	7.20	9.29	3.99	0.31
Session 10	6.58	6.99	9.23	8.57	0.24

*FASA* Family Accommodation Scale – Anxiety, *SCAS* Spence Child Anxiety Scale. SCAS and FASA where not significantly correlated at any session (*p* > .05). Autocorrelation for FASA was (ρ = .24) and for SCAS (ρ = .17)

**Fig. 1:**
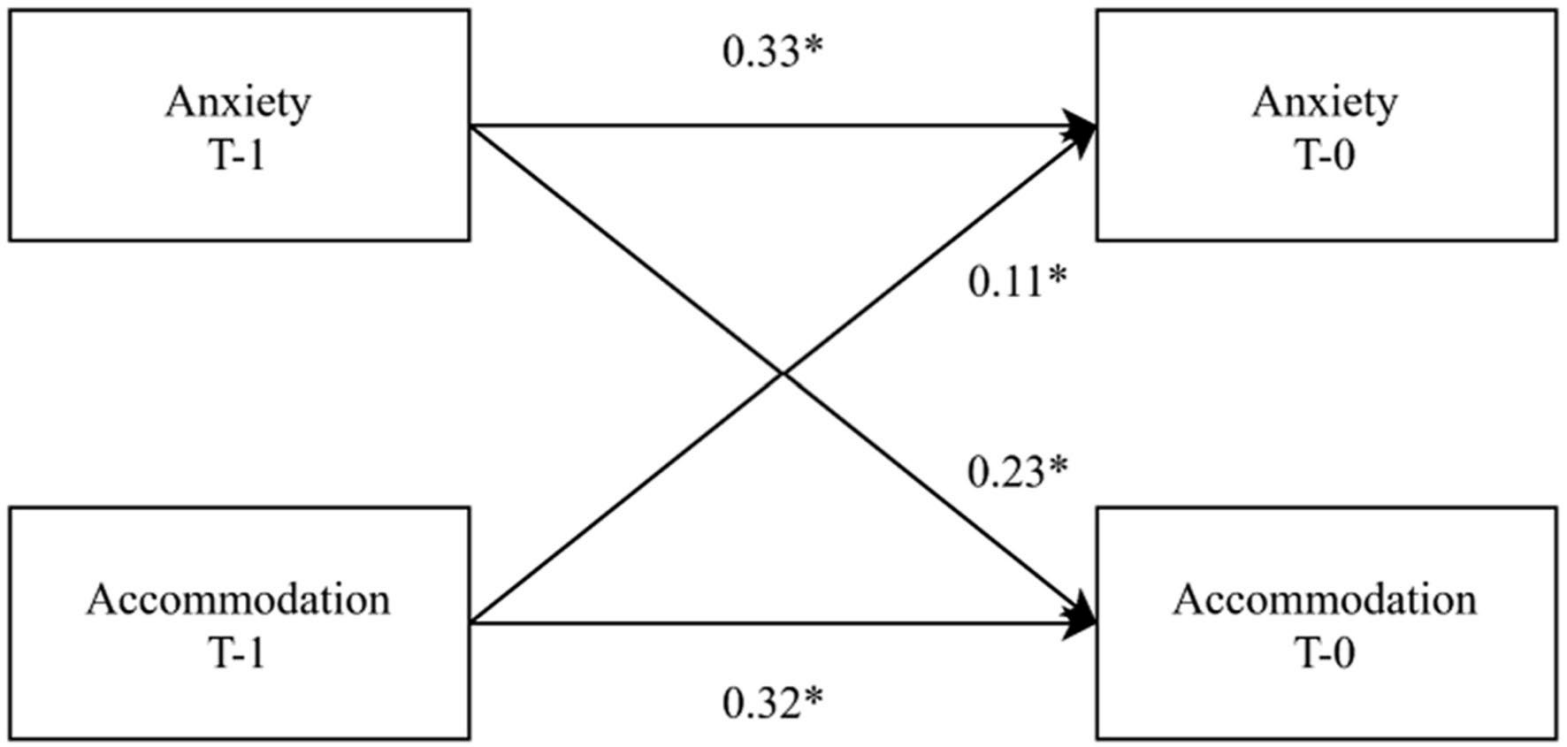
Previous session (T-1) to current session (T-0) relationship between variables. Anxiety is measured by Spence Child Anxiety Scale. Accommodation is measured by Family Accommodation Scale-Anxiety. Coefficients are standardized to describe within-person change. The cross-lagged effect of accommodation on anxiety was substantially greater than the reverse cross-lagged effect. *Denotes coefficients where the posterior 95% credibility interval did not overlap 0

The multivariate cross-lagged model of family accommodation and youth anxiety symptoms is shown in Fig. 1. The results of this model are further described in Table 2. Figure 1 shows the model where previous sessions FASA and SCAS are used to predict the current session’s FASA and SCAS. Standardized coefficients for the model are displayed in Table 2.

**Table 2:** Regression coefficients for the cross-lagged model

	*β*	95% CI	
		LL	UL
Fixed effects			
Slopes			
FASA → SCAS^a^	0.11	0.06	0.17
SCAS → FASA^b^	0.23	0.08	0.38
Autoregression			
SCAS	0.33	0.23	0.42
FASA	0.32	0.23	0.41
Between person variance			
Slopes			
FASA → SCAS	0.05	0.00	0.13
SCAS → FASA	0.32	0.12	0.50
Autoregression			
SCAS	0.13	0.01	0.25
FASA	0.12	0.01	0.22

*FASA* Family Accommodation Scale—Anxiety, *SCAS* Spence Child Anxiety Scale, *CI* Credibility interval, *LL* lower limit, *UL* upper limit. The model describes the cross-lagged model with variables from previous session. All measurements were person-mean centered

^a^Denotes the cross-lagged effect of FASA on SCAS

^b^Denotes the cross-lagged effect of SCAS on FASA

The cross-lagged within-person standardized model answers the question: “does family accommodation predict next session anxiety more than anxiety predicts next session family accommodation?”. This model was a good fit for predicting SCAS (*R*^*2*^ = 0.26) and FASA (*R*^*2*^ = 0.30). In this study, the standardized effect of family accommodation, from the previous session, on anxiety during this session was 0.11, 95% CI [0.06, 0.17], whereas the standardized effect of anxiety, from the previous session, on accommodation during this session was 0.23, 95% CI [0.08, 0.38]. The difference between the two cross-lagged effects was substantial (posterior probability of difference = 0.91), suggesting that from session to session, youth anxiety increases accommodation *more* than accommodation increases youth anxiety. The cross-lagged effects were consistent across individuals, with the model showing small amounts of between-person differences in effects (ICC = 0.10). Thus, for a subset of individuals, there was an opposite influence of cross-lagged effects, such that for 3% of the sample a higher amount of accommodation led to fewer anxiety symptoms, and for 7% of the sample higher anxiety led to less accommodation.

## Discussion

We investigated the directional relationship between family accommodation and youth anxiety during active treatment using multivariate multilevel cross-lagged autoregressive models. Results indicate a bidirectional relationship between family accommodation and youth anxiety, with anxiety having a stronger influence over family accommodation than the reverse from session to session. This means that to some extent, less family accommodation in the previous session predicted fewer anxiety symptoms in the current session, but to a greater extent, fewer anxiety symptoms in the previous session predicted less family accommodation in the current session. These findings were consistent across individuals, with small amounts of between-person variance. These findings imply that interventions aimed at reducing anxiety symptoms should address *both* family accommodation and youth anxiety for maximum effectiveness. While reducing both family accommodation and youth anxiety should result in a positive feedback loop, focusing on just one of these could impede therapeutic progress.

In order to assess the ability to generalize from the present study, it is of importance to address certain characteristics of the sample related to age and diagnostic profile. The present sample contains youth that are considerably older (*M* = *15.4,* SD = 1.4) compared to other studies of family accommodation and youth anxiety [17, 20, 31]. Differences in age may limit the generalizability of findings. However, previous research has not clarified how age affects the relationship between family accommodation and youth anxiety. Some studies suggest that higher age increases the amount of accommodation [17] others that it decreases [20], whereas others have found no relation [31]. Thus, further research is needed to explore whether the present findings are similar in younger children.

Another important aspect of the current sample that might affect the ability to generalize from findings is that the majority of participants had social phobia as their primary diagnosis (69.9%). This particular aspect of the sample may potentially limit the generalizability of findings. However, existing research suggests that the presence of social phobia does not have a significant effect on family accommodation compared to the effect of accommodation on other diagnoses [20, 31]. Conversely, the presence of separation anxiety disorder has been suggested to significantly increase levels of family accommodation [11, 31]. Thus, the present results may not generalize to separation anxiety disorder, which affects family accommodation differently from other anxiety disorders.

It is also important to address aspects of the treatment delivered that may have affected results. The treatment was delivered in small groups, with a high degree of parental involvement and consisted of 35 h of treatment with individual sessions lasting up to 5 h. This format is different from CBT for anxiety in youth offered in many settings. CBT for youth anxiety is typically delivered individually or in larger groups compared to the present study, and lasts on average 18 h with shorter sessions compared to the present study [22]. The length of treatment and type of involvement would be suspected to reduce parental anxiety and distress, which may be associated with lower levels of accommodation [41, 42]. Parental anxiety has also been found to be associated with youth anxiety [21], and may thus be an important confounding factor explaining the directional relationship between family accommodation and youth anxiety. However, other studies have not found an association between parental distress and family accommodation [12, 43]. Thus, it is uncertain to what degree the findings in the present study are confounded by changes in parent mental health.

Within the abovementioned caveats to the ability to generalize from findings the results of the cross-lagged model indicate that from session to session there is a bidirectional relationship between family accommodation and anxiety, with anxiety being more influential. This means that to an extent reduction in family accommodation preceded reduction in youth anxiety, but to a greater extent reduction in youth anxiety preceded reduction in family accommodation. This finding agrees with previous research that suggests that CBT delivered to youth may also indirectly affect parents [21, 44]. Such findings are important for the ongoing development of parent-only interventions focusing on family accommodation [15, 23]. On one hand, our findings support such parent-only intervention by showing that family accommodation can be targeted as an intervention for youth anxiety. On the other hand, our findings indicate that parent-only approaches are suboptimal since our findings indicate that the most beneficial approach is to target both family accommodation and youth anxiety. Furthermore, our findings showed that direct reduction of youth anxiety was more influential than family accommodation, suggesting that family involvement should be seen as an addition to, and not a substitute for, CBT for youth anxiety.

Certain limitations to the current study should be noted. First, the FASA represented a combination of father and mother reports and cannot differentiate between father- and mother-rated family accommodation. Although the inter-rater agreement between parents was high (Cronbach’s α = 0.82), it is possible that a larger sample with reports from both fathers and mothers would find a difference in effect between the two. Second, the directional relationship was assessed only from session to session, and it may be the case that the effects change at larger intervals. Third, the sample consisted of ethnic Norwegian youth undergoing treatment for anxiety, and findings may not generalize to other settings. Notwithstanding these limitations, our study provides a needed empirical investigation of the theoretical directional relationship between family accommodation and youth anxiety at the individual level. The findings constitute important and novel evidence for the relationship between family accommodation and youth anxiety and underscore the importance of targeting both factors when intervening to reduce youth anxiety. Future research should extend these findings to longer periods, as the importance of addressing family accommodation may be more visible over longer periods. In addition, future research utilizing randomization should be performed to investigate the causal relationships between family accommodation and youth anxiety during treatment.

## Summary

Family accommodation has recently received increasing attention as a promising target for interventions against youth anxiety. The premise for such interventions is that increased levels of family accommodation precede increases in youth anxiety and therefore changes in family accommodation can attenuate the effect of psychotherapy. However, this assumption has not been tested, at it may be equally reasonable to assume that changes in youth anxiety precede changes in family accommodation. A cross-lagged cross-panel design was used to assess accommodation and anxiety for 10 sessions for 73 youths with an anxiety disorder, who were receiving cognitive-behavioral therapy. The findings from the present study suggest that there is a bidirectional relationship between family accommodation and youth anxiety, for youth undergoing treatment for anxiety disorders. The findings indicate that both family accommodation and youth anxiety should be addressed in interventions targeting youth anxiety. The findings on the directional relationship between family accommodation and youth anxiety indicate that the direct reduction of youth anxiety should be prioritized over the reduction of family accommodation.
